# Syndromic Deafness Gene *ATP6V1B2* Controls Degeneration of Spiral Ganglion Neurons Through Modulating Proton Flux

**DOI:** 10.3389/fcell.2021.742714

**Published:** 2021-10-21

**Authors:** Shiwei Qiu, Weihao Zhao, Xue Gao, Dapeng Li, Weiqian Wang, Bo Gao, Weiju Han, Shiming Yang, Pu Dai, Peng Cao, Yongyi Yuan

**Affiliations:** ^1^Department of Otolaryngology, Head and Neck Surgery, Institute of Otolaryngology, Genetic Testing Center for Deafness, Chinese PLA General Hospital; National Clinical Research Center for Otolaryngologic Diseases; Key Lab of Hearing Impairment Science of Ministry of Education; Key Lab of Hearing Impairment Prevention and Treatment of Beijing, Beijing, China; ^2^The Institute of Audiology and Balance Science, Artificial Auditory Laboratory of Jiangsu Province, Xuzhou Medical University, Xuzhou, China; ^3^Department of Otolaryngology General Hospital of Tibet Military Region, Lhasa, China; ^4^Department of Otolaryngology, PLA Rocket Force Characteristic Medical Center, Beijing, China; ^5^Department of Neurobiology, School of Basic Medical Sciences, Beijing Key Laboratory of Neural Regeneration and Repair, Advanced Innovation Center for Human Brain Protection, Capital Medical University, Beijing, China; ^6^National Institute of Biological Sciences, Beijing, China

**Keywords:** syndromic hearing loss, *Atp6v1b2*, lysosome, apoptosis, function compensation

## Abstract

*ATP6V1B2* encodes the V1B2 subunit in V-ATPase, a proton pump responsible for the acidification of lysosomes. Mutations in this gene cause DDOD syndrome, DOORS syndrome, and Zimmermann–Laband syndrome, which share overlapping feature of congenital sensorineural deafness, onychodystrophy, and different extents of intellectual disability without or with epilepsy. However, the underlying mechanisms remain unclear. To investigate the pathological role of mutant *ATP6V1B2* in the auditory system, we evaluated auditory brainstem response, distortion product otoacoustic emissions, in a transgenic line of mice carrying c.1516 C > T (p.Arg506^∗^) in *Atp6v1b2, Atp6v1b2*^Arg506*/Arg506*^. To explore the pathogenic mechanism of neurodegeneration in the auditory pathway, immunostaining, western blotting, and RNAscope analyses were performed in Atp6v1b2^Arg506*/Arg506*^ mice. The Atp6v1b2^Arg506*/Arg506*^ mice showed hidden hearing loss (HHL) at early stages and developed late-onset hearing loss. We observed increased transcription of *Atp6v1b1* in hair cells of Atp6v1b2^Arg506*/Arg506*^ mice and inferred that *Atp6v1b1* compensated for the *Atp6v1b2* dysfunction by increasing its own transcription level. Genetic compensation in hair cells explains the milder hearing impairment in Atp6v1b2^Arg506*/Arg506*^ mice. Apoptosis activated by lysosomal dysfunction and the subsequent blockade of autophagic flux induced the degeneration of spiral ganglion neurons and further impaired the hearing. Intraperitoneal administration of the apoptosis inhibitor, BIP-V5, improved both phenotypical and pathological outcomes in two live mutant mice. Based on the pathogenesis underlying hearing loss in *Atp6v1b2*-related syndromes, systemic drug administration to inhibit apoptosis might be an option for restoring the function of spiral ganglion neurons and promoting hearing, which provides a direction for future treatment.

## Introduction

V-ATPase is a multi-subunit enzyme complex also known as the vacuolar H^+^-ATPase. This proton pump is mainly responsible for the acidification of lysosomes and other membrane-bound compartments (Beyenbach and Wieczorek, 2006). V-ATPase comprises a peripheral V1 domain catalyzing ATP hydrolysis and a membrane integral V0 domain involved in proton translocation (Beyenbach and Wieczorek, 2006; Mindell, 2012). The V1 domain includes at least eight different subunits (A-H), while the V0 domain includes six different subunits (a,d,e,c,c′, and c″). The loss of any subunit will disrupt the assembly of V-ATPase and affect lysosome acidification, which could lead to various disorders (Ma et al., 2011).

*ATP6V1B2*, which encodes the V1B2 subunit in V-ATPase, is the causative gene for dominant deafness-onychodystrophy syndrome (DDOD syndrome, MIM: 124480) (Yuan et al., 2014); deafness, onychodystrophy, osteodystrophy, intellectual disability, and seizures syndrome (DOORS syndrome, MIM: 220500) (Beauregard-Lacroix et al., 2020); and Zimmermann–Laband syndrome (ZLS, MIM: 135500) (Kortum et al., 2015). These syndromes share the symptoms of congenital sensorineural deafness, onychodystrophy, and different extents of intellectual disability without or with epilepsy. *ATP6V1B2* c.1516 C > T, p.Arg506^∗^, was identified in all families with DDOD syndrome (Yuan et al., 2014; Menendez et al., 2017). To investigate the pathological role of mutant *ATP6V1B2* in the neurosensory system, we generated a transgenic line of mice carrying c.1516 C > T (p.Arg506^∗^) in *Atp6v1b2, Atp6v1b2*^Arg506*/Arg506*^ (homozygous mutant), and identified that the mutant mice displayed obvious cognitive defects for which impairment in the hippocampal CA1 region might be the pathological basis (Zhao et al., 2019). The interaction between the V1B2 and V1E subunits was found to be weaker in Atp6v1b2^Arg506*/Arg506*^mice than in wild-type (WT) mice, indicating that the assembly of V-ATPase was affected by the mutation (Zhao et al., 2019). In another prior study, when pIRES2-EGFP-*ATP6V1B2* WT and pIRES2-EGFP-*ATP6V1B2* c.1516 C > T mutant plasmids were transfected into HEK293 cells, the lysosomal pH was increased, revealing the reduced acidification in the lysosome caused by the c.1516 C > T mutation (Yuan et al., 2014). Lysosomes, as scavengers of living cells, play a critical role in cellular metabolism. An abnormal lysosomal pH will cause dysfunctional macromolecule degradation and lead to lysosomal storage diseases. Loss of the V-ATPase subunits in the *Drosophila* fat body cells resulted in an abnormal pH in the lysosomal lumen, causing an accumulation of non-functional lysosomes and leading to a blockade of autophagic flux (Mauvezin et al., 2015). Additionally, abnormalities in autophagy caused by V-ATPase defects are associated with neurodegenerative diseases (Peric and Annaert, 2015; Cerri and Blandini, 2019).

Whether the *Atp6v1b2* c.1516 C > T mutation results in autophagic dysfunction and leads to abnormal auditory function remains unanswered. However, the Atp6v1b2^Arg506*/Arg506*^ mice displayed normal auditory brainstem response (ABR) thresholds before 24 weeks of age (Zhao et al., 2019), while in the patients with *ATP6V1B2-*related syndromes, the hearing loss is congenital and severe. Genetic compensation can be induced by non-sense mutations that could occur between homologous genes (Ma et al., 2019). Two highly homologous genes encode V1B subunits: *ATP6V1B1* and *ATP6V1B2*. The B2 subunit of V-ATPase was shown to functionally substitute for the B1 subunit (Paunescu et al., 2007). Therefore, we speculate that genetic compensation might be responsible for the phenotype of Atp6v1b2^Arg506*/Arg506*^ mice.

Herein, we performed long-term studies on hearing in Atp6v1b2^Arg506*/Arg506*^ mice, investigated the pathogenic mechanism of neurodegeneration in the auditory pathway, and confirmed the genetic compensation in Atp6v1b2^Arg506*/Arg506*^ mice to explain the difference between hearing impairments observed in patients and those produced in this mouse model.

## Materials and Methods

### Animals

Atp6v1b2^Arg506*/Arg506*^mice were generated in the C57BL/6 strain background by Shanghai Model Organisms Center, Inc., (Shanghai, China), as described in detail previously (Zhao et al., 2019). Heterozygous (HE) mutant mice were crossed to generate homozygous Atp6v1b2^Arg506*/Arg506*^ (HO) mutant mice, and WT littermates were used as controls. Male WT and Atp6v1b2^Arg506*/Arg506*^ (HO) mutant mice were tested.

### Bax Inhibitor Peptide V5 Treatment

The concentration of Bax inhibitor peptide V5 (BIP-V5) was adjusted to 100 μmol/L with saline. Starting from 4 weeks after birth, mice (*n* = 8) were injected intraperitoneally at a dose of 10 μL/g body weight once weekly until 40 weeks after birth. In addition, WT (*n* = 8) and Atp6v1b2^Arg506*/Arg506*^ (*n* = 8) control mice were injected intraperitoneally with saline alone.

### ABR Analysis

Hearing was evaluated in WT and Atp6v1b2^Arg506*/Arg506*^ mice at the age of 4, 12, 20, 28, 36, and 40 weeks (*n* = 6 per genotype and age group). In brief, the stimuli of ABR included click and tone-burst (1, 2, 4, 8, 16, 24, and 32 kHz) and were presented from 90 to 10 dB SPL. Amplitude and latency of ABR wave I were analyzed for 90 dB SPL click stimuli.

### Immunostaining

Inner ear tissues were dissected and fixed in 4% paraformaldehyde for 1–2 h at room temperature or for 12 h at 4°C, followed by decalcification in 10% EDTA (pH = 7.2) at 4°C for 5 days. For cryosections, the calcium-depleted cochlea tissues were placed into a 30% sucrose solution for dehydration. Serial sections were cut at 10 μm thickness. For basilar membrane with organ of Corti, cochlea ducts were dissected.

The primary antibodies used were as follows: rabbit anti-Myo7a (Proteus Biosciences, Ramona, CA, United States, 1:300), mouse anti-Ctbp2 (BD Biosciences, San Jose, CA, United States, 1:200), chicken anti-NFH (AB5539, EMD Millipore, Billerica, MA, United States, 1:500), rabbit anti-beta III tubulin (ab230847, Abcam, United Kingdom, 1:500), rabbit anti-cleaved caspase-3 (9661, Cell Signaling Technology, Boston, MA, United States, 1:500), rat anti-MBP (MAB386, EMD Millipore, Billerica, MA, United States, 1:500), rabbit anti-caspase-3 (ab4051, Abcam, United Kingdom, 1:500), rat anti-LAMP1 (ab25245, Abcam, United Kingdom, 1:500), rabbit anti-Bax (ab199677, Abcam, United Kingdom, 1:500), rabbit anti-BCL2 (12789-1-AP, Proteintech, Chicago, IL, United States, 1:500), rabbit anti-LC3B (ab48394, Abcam, United Kingdom, 1:500), rabbit anti-TOM20 (SC-11415, CiteAB, Santa Cruz, CA, United States, 1:500), and mouse anti-cytochrome C (556432, BD Pharmingen, United States, 1:500). The secondary antibodies used were as follows: goat anti-rabbit IgG (A-11008, Thermo Fisher Scientific, Waltham, MA, United States, 1:500), goat anti-mouse IgG (A-10684, Thermo Fisher Scientific, Waltham, MA, United States, 1:500), goat anti-rat IgG (A-11077, Thermo Fisher Scientific, Waltham, MA, United States, 1:500), and goat anti-chicken IgY (A-21449, Thermo Fisher Scientific, Waltham, MA, United States, 1:500). Nuclei were labeled with DAPI.

### Confocal Laser Scanning Microscopy

Confocal *z*-stacks (0.3 μm step size) of cochlea tissues were taken using either a Zeiss LSM800 or a Leica SP8 microscope. ImageJ software (version 1.46, NIH, MD, United States) was used for image processing of *z*-stacks. All immunofluorescence images shown in this study are representative of at least three individual mice in each group.

### Western Blotting

The cochlea tissues were quickly removed from mice and cryo-milled in RIPA lysis buffer and centrifuged. The supernatants were collected, and the protein concentration was detected using the BCA protein assay kit (PI23227, Thermo Fisher Scientific, Waltham, MA, United States). Then equal amounts of protein sample were separated by 12% Tris/Glycine SDS-PAGE and transferred to a polyvinylidene difluoride membrane. The primary antibodies used were the same as those used for immunostaining analysis. The experiments were independently repeated three times.

### Transmission Electron Microscopy

Mice were perfused intracardially with 4% paraformaldehyde (in 0.1 M phosphate buffer). Cochleae were then isolated and postfixed with 1.5% paraformaldehyde and 2.5% glutaraldehyde, followed by decalcification in 10% EDTA at 4°C for 5 days and osmification in 1% osmium tetroxide. Then the samples were dehydrated in ethanol and embedded in araldite resin. After resin solidification, semi-thin sections were made stained for localization. Then ultra-thin sections, uranium dioxide acetate saturated and lead citrate were stained respectively. Transmission electron microscopy (TEM) was performed using a Jeol 1400-plus electron microscope (JEM-1400, JEDL, Tokyo). Multiple non-overlapping regions of the ANF cross-sections were imaged at ×3,400 magnification. All images of semi-thin sections and electron microscope sections shown are representative of at least three individual mice in each group.

### RNAscope

Cochleae of 4-week-old mice were perfused and decalcified. After freezing the tissues in OCT compound with dry ice or liquid nitrogen, they were stored in an airtight container at –80°C. Before tissue sectioning, the tissue blocks were placed at –20°C for at least 1 h in a cryostat. The blocks were then sectioned to 7 μm. After air drying the slides for 20 min at –20°C, the RNAscope experiment was performed as previously described (Shrestha et al., 2018; Sun et al., 2018). The following probes for RNA binding were used: Mm-Atp6v1b1 (#804281, Advanced Cell Diagnostics, United States) and Mm-Atp6v1b1-C2 (#804291-C2, Advanced Cell Diagnostics). The sections were imaged using a confocal microscope.

### Statistical Analysis

Statistical analyses were performed using Microsoft Excel and SPSS (18.0). Statistical differences were analyzed using *t*-test. The data are represented as the mean ± SEM. Densitometric analysis in the western blotting experiment was performed using the ImageJ software. *P* values less than 0.05 were considered to indicate statistical significance.

## Results

### Atp6v1b2^Arg506*/Arg506*^ Mice Begin to Develop Hidden Hearing Loss at 12 Weeks and Develop Hearing Loss at 28 Weeks After Birth

**FIGURE 1:**
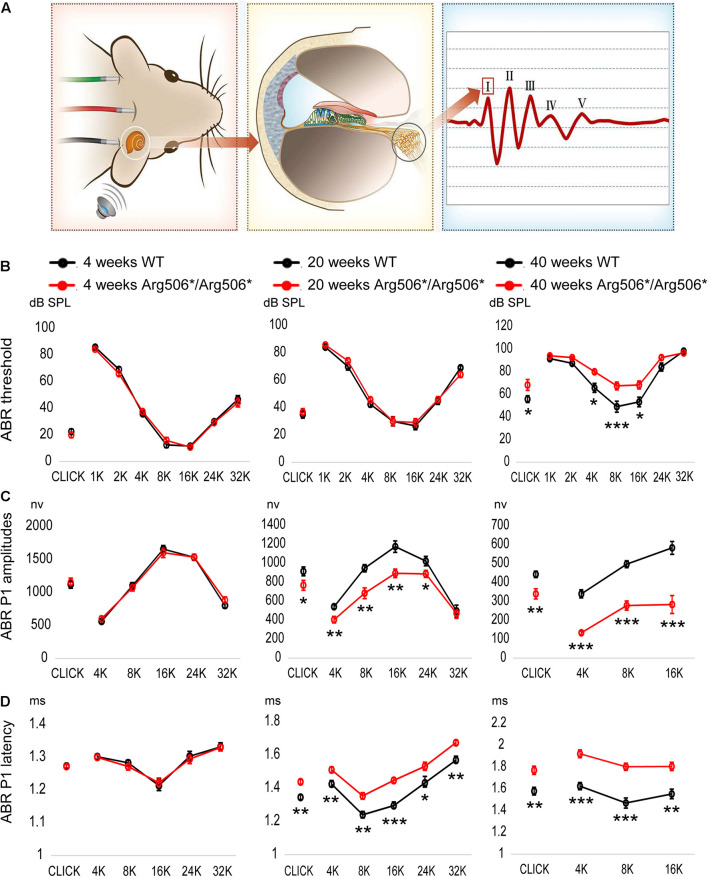
Progressive hearing impairment with increasing age in *Atp6v1b2*^Arg506*/Arg506*^ mice. **(A)** Auditory brainstem responses (ABR) were recorded to evaluate the hearing in mice. After acoustic stimulation, the recording electrode recorded five waveforms, of which peak I (P1) was derived from spiral ganglion neurons in the mouse cochlea. The hearing thresholds were defined as the lowest sound intensity that elicited identifiable waves. **(B)** ABR thresholds (shown in ordinate) to click and to 1, 2, 4, 8, 16, 24, and 32 kHz (shown in abscissa) tone-burst stimuli were compared between WT and *Atp6v1b2*^Arg506*/Arg506*^ mice. At 4 weeks and 20 weeks, ABR thresholds showed no significant difference between WT and *Atp6v1b2*^Arg506*/Arg506*^ mice. At 40 weeks, ABR thresholds to click and to 4, 8, and 16 kHz stimuli were significantly higher in *Atp6v1b2*^Arg506*/Arg506*^ than in WT mice. **(C)** ABR P1 amplitudes (shown in ordinate) were compared between WT and *Atp6v1b2*^Arg506*/Arg506*^ mice. There was no significant difference at 4 weeks. At 20 weeks, *Atp6v1b2*^Arg506*/Arg506*^ mice had significantly lower peaks to click and to 4, 8, 16, and 24 kHz tone-burst stimuli than WT mice. At 40 weeks, *Atp6v1b2*^Arg506*/Arg506*^ mice had significantly lower peaks to click and to 4, 8, and 16 kHz tone-burst stimuli than WT mice. **(D)** ABR P1 latencies (shown in ordinate) stimulated by different sound types (shown in abscissa) with the same sound intensity (90 dB SPL) were compared between WT and *Atp6v1b2*^Arg506*/Arg506*^ mice. There was no significant difference at 4 weeks. At 20 weeks, *Atp6v1b2*^Arg506*/Arg506*^ mice showed significantly longer latencies to click and to 4, 8, 16, 24 and 32 kHz tone-burst stimuli than WT mice. At 40 weeks, *Atp6v1b2*^Arg506*/Arg506*^ mice showed significantly longer latencies to click and to 4, 8, and 16 kHz tone-burst stimuli than WT mice. The *t*-test was performed to evaluate statistical significance; n represents the number of test ears, *n* = 6 for each group; * denotes *P* < 0.05, ** denotes *P* < 0.01, *** denotes *P* < 0.001. Data are described as mean +SEM (standard error of mean).

Mice were phenotyped using ABR at 4–40 weeks of age. The ABR waveform in mice is composed of five peaks, corresponding to electrical signals generated by different components of the peripheral and central auditory pathway (Figure 1A). The ABR at 4, 20, and 40 weeks, which represent the early, middle, and aged stages of Atp6v1b2^Arg506*/Arg506*^ mice, are shown in Figures 1B–D. The ABR of the mice at other ages are shown in Supplementary Figures 1A–C. The thresholds for Atp6v1b2^Arg506*/Arg506*^ mice did not differ significantly from those of WT mice at 4, 12, and 20 weeks. Atp6v1b2^Arg506*/Arg506*^ mice presented hidden hearing loss (HHL) at 12 and 20 weeks with lower P1 amplitudes and a longer latency than WT mice (Figures 1C,D and Supplementary Figures 1B,C). From 28 weeks, Atp6v1b2^Arg506*/Arg506*^ mice showed progressive HL and increased ABR thresholds compared with WT mice (70 dB SPL average, Supplementary Figure 1A). However, DPOAE threshold of 4–32 kHz showed no significant difference between Atp6v1b2^Arg506*/Arg506*^ (4 kHz: 41.67 ± 3.33, 8 kHz: 38.33 ± 1.67, 16 kHz: 43.33 ± 3.33, 32 kHz: 65 ± 5.77, *n* = 3) and WT mice (4 kHz: 40 ± 2.89, 8 kHz: 38.33 ± 4.41, 16 kHz: 45 ± 5.77, 32 kHz: 68.33 ± 3.33, *n* = 3) (*T*-test, *P* > 0.05, Supplementary Figure 1D) at 6 months, which indicated the normal function of outer hair cells (OHCs).

**FIGURE 2:**
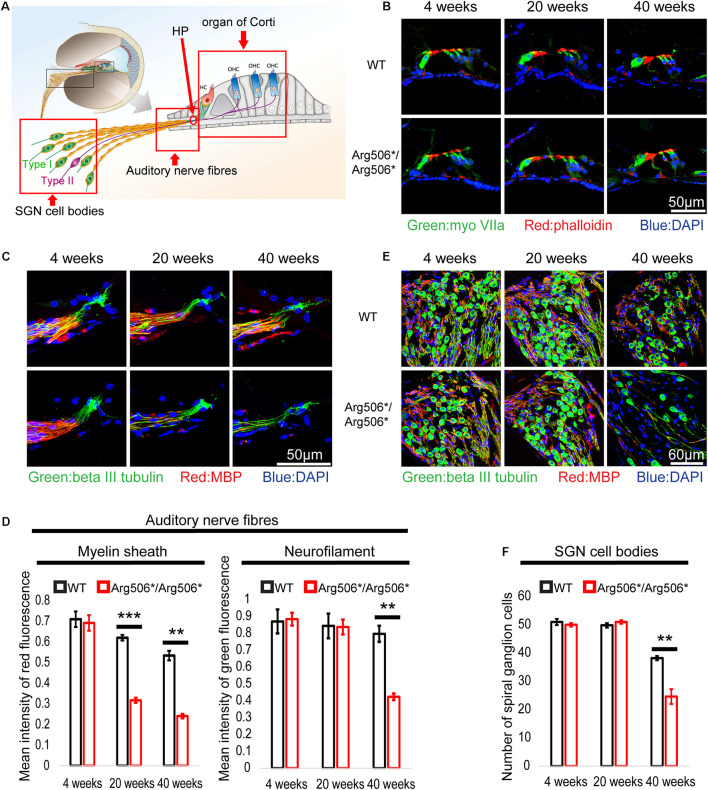
Abnormal morphology in the cochlea of *Atp6v1b2*^Arg506*/Arg506*^ mice at different postnatal ages of 4, 20, and 40 weeks. **(A)** Illustration of the cochlear architecture. The three areas within the red frame (from left to right): spiral ganglion neuron (SGN) cell bodies, auditory nerve fibers, and organ of Corti. **(B)** There is no obvious difference in the morphology of organ of Corti between WT and *Atp6v1b2*^Arg506*/Arg506*^ mice. Phalloidin, MyoVIIa, and DAPI are markers of hair cell cilia, inner/outer hair cells, and nuclei, respectively. The experiments were repeated three times. **(C)** Representative images of auditory nerve fibers for WT and *Atp6v1b2*^Arg506*/Arg506*^ mice. **(D)** Statistical results of the red and green fluorescence intensities for panel **(C)**. The intensity of red fluorescence (myelin) decreased significantly at 20 and 40 weeks in *Atp6v1b2*^Arg506*/Arg506*^ mice. The intensity of green fluorescence (nerve fibers) decreased significantly at 40 weeks in *Atp6v1b2*^Arg506*/Arg506*^ mice. **(E)** Representative images of SGN cell bodies of WT and *Atp6v1b2*^Arg506*/Arg506*^ mice. **(F)** Statistical results of the number of SGN cell bodies for panel **(E)**. The number of SGN cell bodies decreased significantly in *Atp6v1b2*^Arg506*/Arg506*^ mice compared to that in WT mice at 40 weeks. The myelin sheath, nerve fibers, and nuclei are labeled with red (MBP), green (beta III tubulin), and blue (DAPI) fluorescence, respectively. The *t*-test was performed to evaluate the statistical significance. Each group included three mice, and one slice for each mouse was processed. ** denotes *P* < 0.01, *** denotes *P* < 0.001. Data are described as mean ± SEM (standard error of mean). HP, habenula perforate.

No morphological changes of the organ of Corti between WT and Atp6v1b2^Arg506*/Arg506*^ mice were observed (Figure 2B), also indicating that hearing loss in Atp6v1b2^Arg506*/Arg506*^ mice was not related to hair cell loss. The synaptic density in Atp6v1b2^Arg506*/Arg506*^ mice did not differ significantly from that in WT mice at different ages (Supplementary Figure 2). Interestingly, although the auditory nerve fibers of Atp6v1b2^Arg506*/Arg506*^ mice did not change significantly at 4 weeks, the myelin sheath covering the auditory nerve fibers decreased at 20 weeks (Figures 2C,D). We labeled the unmyelinated nerve fibers in the osseous spiral lamina (OSL) of mouse cochlea with the NFH antibody (Supplementary Figures 3A–F). In the cochlea of Atp6v1b2^Arg506*/Arg506*^ mice at 20 weeks, parts of auditory nerve fibers in the OSL stained positive for NFH, indicating the presence of auditory nerve fiber demyelination (Supplementary Figures 3C,F), which was consistent with myelin stain results (Figures 2C,D). To further clarify the changes in myelination of the nerve fibers, we performed TEM to observe the morphology of the myelin sheath in mice at 4, 12, and 20 weeks of age (Supplementary Figures 3G–L). Atp6v1b2^Arg506*/Arg506*^ mice showed vacuolar-like changes in the myelin sheath of auditory nerve fibers in the OSL at 4 weeks, but the myelin sheath was still wrapped around the nerve fibers (Supplementary Figure 3J). Demyelination was observed in the auditory nerve fibers of Atp6v1b2^Arg506*/Arg506*^ mice at 12 and 20 weeks (Supplementary Figures 3K,L). These changes did not appear in WT mice (Supplementary Figures 3G–I). The above results indicate that demyelination of cochlear auditory nerve fibers was the pathological basis for HHL in Atp6v1b2^Arg506*/Arg506*^ mice.

In addition, the number of SGNs decreased significantly in Atp6v1b2^Arg506*/Arg506*^ mice compared to that in WT mice at 40 weeks (Figures 2E,F). The excitation of type I SGNs is caused by the action potential of IHCs and is further transmitted to the neuronal body, type II SGNs connect the OHCs and are mainly responsible for receiving signals from the OHCs (Figure 2A). Another special feature of type II SGNs is that the cell bodies and the nerve fibers that connect the OHCs are unmyelinated. By labeling type II nerve fibers with NFH antibody, we found that the number of type II nerve fibers connecting the OHCs was reduced in Atp6v1b2^Arg506*/Arg506*^ mice at 28 weeks (Supplementary Figures 1E,F). In summary, the decrease in SGNs was the cause of hearing loss in the aged Atp6v1b2^Arg506*/Arg506*^ mice.

### *Atp6v1b2* c.1516C > T Causes Abnormal Autophagy, Which Leads to Apoptosis of the SGNs in Atp6v1b2^Arg506*/Arg506*^ Mice

**FIGURE 3:**
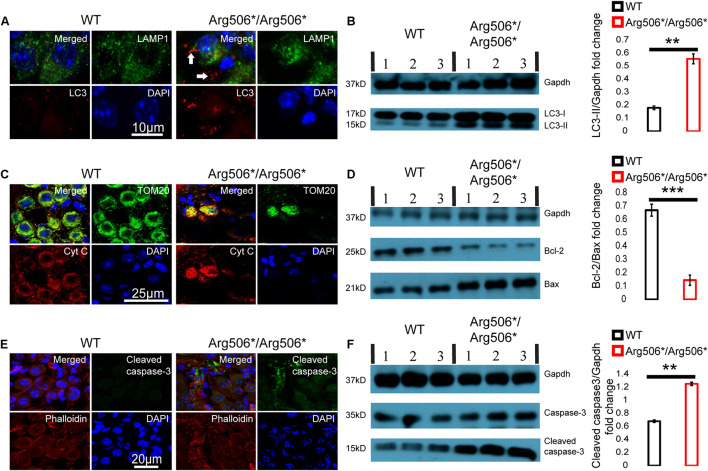
Abnormal autophagy and apoptosis activated by cytochrome C in the spiral ganglion neurons (SGNs) of *Atp6v1b2*^Arg506*/Arg506*^ mice. **(A)** Representative images of immunostaining showed that autophagosomes labeled with LC3 (white arrows) were present in large numbers as dots in the SGNs of *Atp6v1b2*^Arg506*/Arg506*^ mice. **(B)** Left: Western blotting results showed that LC3-II increased in the cochlea of *Atp6v1b2*^Arg506*/Arg506*^ mice compared with that in the cochlea of WT mice. LC3-II can be used to estimate the extent of autophagy. Right: Statistical results of gray value for western blotting. The ratio of LC3-II/Gapdh in the cochlea of *Atp6v1b2*^Arg506*/Arg506*^ mice was significantly higher than that in the cochlea of WT mice (** denotes *P* < 0.01 by *t*-test; *n* = 3 for each group). **(C)** The release of cytochrome C from the mitochondria into the cytosol of the SGNs in *Atp6v1b2*^Arg506*/Arg506*^ mice is shown. Mitochondria are labeled with TOM20 (green). Cytochrome C is labeled as red. In WT mice, cytochrome C remains in the mitochondria and no red signal was observed in the cytosol of the SGNs. In *Atp6v1b2*^Arg506*/Arg506*^ mice, cytochrome C was observed both in the mitochondria and the cytosol of the SGNs, indicating its release from mitochondria. **(D)** Left: Western blotting results showed that in the cochlea of *Atp6v1b2*^Arg506*/Arg506*^ mice, Bcl-2 decreased significantly compared with that in the cochlea of WT mice. Right: Statistical results of gray value for western blotting (*** denotes *P* < 0.001 by *t*-test; *n* = 3 for each group). **(E)** More pronounced positive signal for cleaved caspase-3 in the region of SGNs of *Atp6v1b2*^Arg506*/Arg506*^ mice was identified. Phalloidin is a high-affinity filamentous actin (F-actin) marker, which is used to mark the cytoskeleton. **(F)** Left: Western blotting results showed that cleaved caspase-3 increased in the cochlea of *Atp6v1b2*^Arg506*/Arg506*^ mice compared with that in the cochlea of WT mice. Right: Statistical results of gray value for western blotting. The ratio of cleaved caspase-3/Gapdh in the cochlea of *Atp6v1b2*^Arg506*/Arg506*^ mice was significantly higher than that in the cochlea of WT mice. ** denotes *P* < 0.01. *n* = 3 for each group. Data are described as mean ± SEM (standard error of mean). Cyt C, cytochrome C.

**FIGURE 4:**
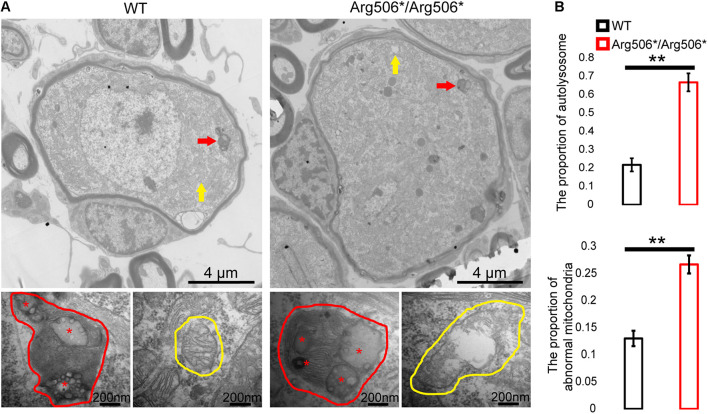
Transmission electron microscopy images of the organelles of SGNs in *Atp6v1b2*^Arg506*/Arg506*^ and WT mice. **(A)** Red arrows and red circles indicate the locations and enlarged views of a typical autophagy lysosome, respectively. Yellow arrows and yellow circles indicate the locations and enlarged views of mitochondria, respectively. In *Atp6v1b2*^Arg506*/Arg506*^ mice, increased degraded substrates indicated by red asterisks in the autophagy lysosomes of the spiral ganglion neurons (SGNs) were observed, and some of the mitochondria appeared swollen. Most of the mitochondria in the SGNs showed normal morphology in WT mice. **(B)** The percentage of autolysosomes and abnormal mitochondria significantly increased in *Atp6v1b2*^Arg506*/Arg506*^ mice. The *t*-test was performed to evaluate statistical significance. The percentage of autolysosomes in lysosomes and the percentage of abnormal mitochondria were manually determined. Each group included three mice, and one slice for each mouse was processed. Data are described as mean ± SEM (standard error of mean). ** denotes *P* < 0.01.

Western blotting results revealed increased LC3-II in the cochlea of Atp6v1b2^Arg506*/Arg506*^mice (Figure 3B), indicating that autophagosomes accumulated in the cytoplasm. In Atp6v1b2^Arg506*/Arg506*^ mice, autophagosomes increased in the SGNs (Figure 3A). However, in the organ of Corti, there was no significant difference between WT and Atp6v1b2^Arg506*/Arg506*^mice (Supplementary Figure 4A). To evaluate if this increase in autophagosomes in the SGNs could be explained by a fusion barrier between autophagosomes and lysosomes or the abnormal degradation of the lysosome, we performed TEM analysis. The results showed high levels of substrates in the autophagic lysosomes of the SGNs in Atp6v1b2^Arg506*/Arg506*^ mice (Figure 4A), indicating that autophagosomes could fuse with the lysosomes. However, the proportion of autolysosomes increased in the SGNs of Atp6v1b2^Arg506*/Arg506*^ mice (Figure 4B), suggesting that the substrates in the lysosomal cavity could not be effectively degraded, which further made it difficult for autophagosomes to enter lysosomes. Thus, we speculated that abnormal lysosomal degradation was the cause of the autophagosome accumulation in the cytoplasm of the SGNs.

Immunofluorescence analysis revealed that cytochrome C was released from the mitochondria to the cytosol of the SGNs of Atp6v1b2^Arg506*/Arg506*^ mice (Figure 3C). Western blotting results showed that in the cochlea of Atp6v1b2^Arg506*/Arg506*^ mice, the ratio of Bcl-2 to Bax was reduced significantly compared with that in WT mice (Figure 3D). Results showed that cleaved caspase-3 in the inner ear of Atp6v1b2^Arg506*/Arg506*^ mice increased significantly (Figure 3F). To further verify the exact area of apoptosis, immunofluorescence staining was performed. The results showed that in the cochlea of Atp6v1b2^Arg506*/Arg506*^ mice, apoptosis was mostly observed in the spiral ganglia (Figure 3E), and no excessive apoptosis was identified in the organ of Corti (Supplementary Figure 4C).

These data suggested that in the inner ear of Atp6v1b2^Arg506*/Arg506*^ mice, disequilibrium of the acidic environment in the lysosomes and autophagosome accumulation in the cytoplasm led to a decrease in the ratio of Bcl-2 to Bax, which further increased the outer membrane permeability of the mitochondria and induced the release of cytochrome C and activation of caspase-3.

### BIP-V5 Improves Phenotypical and Pathological Outcomes in Atp6v1b2^Arg506*/Arg506*^ Mice

During the process of BIP-V5 administration, four mice in the Atp6v1b2^Arg506*/Arg506*^-BIP-V5 group died, thus affecting the statistics of the experimental data. We observed that the hearing threshold was restored in two out of four Atp6v1b2^Arg506*/Arg506*^ mice treated with BIP-V5, and they had better ABR waveforms (Supplementary Figure 5), suggesting a potential role of BIP-V5 in improving the hearing of mutant mice. After BIP-V5 intervention, the expression of Bax (Supplementary Figure 6) and the release of cytochrome C (Supplementary Figure 7C) in the mitochondria of the cochlea were significantly reduced. In addition, immunostaining and western blotting results showed that active caspase-3 (Supplementary Figures 6,7D) in the cochlea of Atp6v1b2^Arg506*/Arg506*^ mice was reduced and the number of SGNs (Supplementary Figure 7A) increased significantly with BIP-V5 intervention, but lysosomal function evaluated by autophagosomes was not significantly changed (Supplementary Figures 6,7B).

### Genetic Compensation Exists in the Hair Cells of Atp6v1b2^Arg506*/Arg506*^ Mice

**FIGURE 5:**
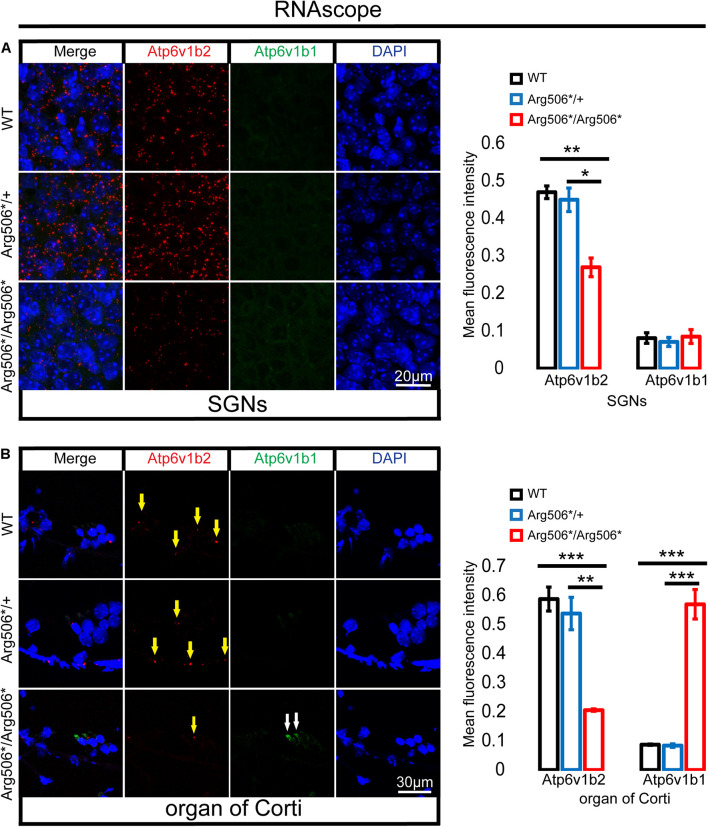
Increased transcription of *Atp6v1b1* occurs in the hair cells of *Atp6v1b2*^Arg506*/Arg506*^ mice. **(A)** Representative images and quantification of RNAscope results in the spiral ganglion neurons (SGNs). The mean fluorescence intensity of *Atp6v1b2* RNA in *Atp6v1b2*^Arg506*/Arg506*^ mice was significantly lower than that in WT and *Atp6v1b2*^Arg506*/+^ mice. No *Atp6v1b1* RNA was found in the SGNs. **(B)** Representative images and quantification of RNAscope results in the organ of Corti. *Atp6v1b2* RNA decreased in *Atp6v1b2*^Arg506*/Arg506*^ mice compared with that in WT and *Atp6v1b2*^Arg506*/+^ mice (yellow arrow). There was no significant difference in the mean fluorescence intensity of *Atp6v1b2* RNA between WT and *Atp6v1b2*^Arg506*/+^ mice. *Atp6v1b1* RNA was found in the hair cells of *Atp6v1b2*^Arg506*/Arg506*^ mice (white arrow) but not in the hair cells of WT and *Atp6v1b2*^Arg506*/+^ mice. *n* = 3 for each group. * denotes *P* < 0.05, ** denotes *P* < 0.01 and *** denotes *P* < 0.001. Data are described as mean ± SEM (standard error of mean).

Clinically, patients diagnosed with *ATP6V1B2*-related syndromes have congenital severe to profound sensorineural hearing loss (Yuan et al., 2014; Menendez et al., 2017; Beauregard-Lacroix et al., 2020); however, the hearing of Atp6v1b2^Arg506*/Arg506*^ mice was quite different. To verify whether functional compensation occurs in the cochlea of Atp6v1b2^Arg506*/Arg506*^ mice, we determined the *Atp6v1b2* and *Atp6v1b1* transcription levels and localization in the cochlea of mice at 4 weeks by RNAscope and found that the RNA level of *Atp6v1b2* was down-regulated in both the SGNs and hair cells in Atp6v1b2^Arg506*/Arg506*^ mice compared with that in WT mice, indicating that the transcription level of *Atp6v1b2* decreased or that the RNA was excessively degraded. In Atp6v1b2^Arg506*/Arg506*^ mice, the transcription level of *Atp6v1b1* significantly increased in the hair cells but not in the SGNs. These results suggested that *Atp6v1b1* may compensate for the loss of *Atp6v1b2* function in the hair cells (Figures 5A,B).

## Discussion

The manifestation of normal auditory thresholds but reduced suprathreshold amplitude of the sound-evoked compound action potential of SGNs, shown as peak I of the ABR waveform, is defined as HHL (Kujawa and Liberman, 2009; Bharadwaj et al., 2014; Mehraei et al., 2016). The pathological mechanisms of HHL are complicated and are not yet fully understood. The electrophysiological results showed that Atp6v1b2^Arg506*/Arg506*^ mice younger than 20 weeks did not have an elevated ABR threshold but had lower P1 amplitudes and longer P1 latencies than WT mice, similar to patients with HHL (Mehraei et al., 2016). Current studies suggest that risk factors associated with HHL include a noisy environment (Liu H. et al., 2019), aging (Fischer et al., 2019), and ototoxic drugs (Liu et al., 2015). LOHL (late-onset hearing loss) is defined as hearing loss that is not present at birth but is identified at a later period. Both environmental and hereditary factors influence the development of LOHL. Clinically, there is no early diagnostic indicator for LOHL until hearing loss is detected. HHL and LOHL are regarded as two diseases with different pathogeneses (Song et al., 2020). We proposed that (1) the Atp6v1b2^Arg506*/Arg506*^ mouse model could be used as a natural model for HHL and LOHL; (2) patients with LOHL may have HHL early in life, and it is important for those who have been diagnosed with HHL to be cautious of risk factors such as noise, ototoxic drugs, etc. to avoid further hearing impairment.

Abnormalities in ribbon synaptic density and demyelination of the auditory nerve fibers have been identified as causes of HHL (Liberman and Kujawa, 2017; Wan and Corfas, 2017). In our study, we identified that HHL in Atp6v1b2^Arg506*/Arg506*^ mice was caused by demyelination of nerve fibers but not the ribbon synaptic density. Schwann cells are essential for the formation of nodes of Ranvier, which is a special structure along the myelinated fibers where voltage-gated sodium channels and potassium channels accumulate for regeneration of action potentials and fast synchronous transmission of electrical signals. This phenomenon explains why nerve fibers with myelin sheaths can conduct action potentials more quickly and efficiently than nerve fibers without myelin sheaths (Rasband and Peles, 2015).

Damage or loss of cells from the auditory pathway in the cochlea, such as IHCs and/or OHCs, supporting cells and SGNs, is a pathological feature of LOHL (Vreugde et al., 2002; Liu et al., 2016; Cheng et al., 2019; Gao et al., 2019; Han et al., 2020; Zhang S. et al., 2020; Zhang Y. et al., 2020; Chen et al., 2021). HCs are highly specialized cells attached to the basement membrane in the organ of Corti in the cochlea, and mainly function in transduce the sound mechanical vibration into the electrical signal (He et al., 2017, 2020, 2021; Tan et al., 2019; Jiang et al., 2020; Zhong et al., 2020; Zhou et al., 2020). These cells have hair-like protrusions (stereocilia) embedded in the tectorial membrane (Wang et al., 2017; Liu Y. et al., 2019; Qi et al., 2019, 2020; Cheng et al., 2021; Fu et al., 2021; Zhang et al., 2021). Incoming soundwaves distort the basement membrane, and the resulting mechanical distortion of the stereocilia is transduced into neural signals that are conveyed through the SGNs to the auditory regions of the brain (Ding et al., 2020; Guo et al., 2020; Jiang et al., 2020; Qian et al., 2020; Liu et al., 2021; Lv et al., 2021; Yuan et al., 2021). Improved culture system of SGNs facilitates the study of physiology and pathophysiology, and promotes identification of potential therapeutic targets for SGNs protection and regeneration (Guo et al., 2016, 2019, 2020; Waqas et al., 2017; Yan et al., 2018; Liu W. et al., 2019; Liu et al., 2021). In this study, Atp6v1b2^Arg506*/Arg506*^ mice developed demyelination of nerve fibers, followed by loss of nerve fibers and SGN cell bodies. This process appears to be neurodegenerative. However, the cochlear hair cells of Atp6v1b2^Arg506*/Arg506*^ mice were morphologically normal.

Hearing loss in patients with DDOD syndrome is primarily treated by cochlear implantation. However, although the implanted cochlea worked well, clinical follow-up of DDOD syndrome patients with cochlear implantation revealed that their language rehabilitation was unsatisfactory. Our findings regarding the pathological changes in SGNs and mild learning and memory problems in DDOD syndrome patients (Zhao et al., 2019) could explain this clinical puzzle.

V-ATPase has two main effects on the organelle: maintaining the acidic environment of the organelles and participating in and regulating the fusion between vesicle-type organelles, such as the fusion between lysosomes and autophagosomes (Colacurcio and Nixon, 2016). We have demonstrated that abnormalities in the assembly of the subunits of V-ATPase occurred in Atp6v1b2^Arg506*/Arg506*^ mice, which affects the transfer of hydrogen ions into the lysosome (Yuan et al., 2014; Zhao et al., 2019). In this study, we observed the presence of metabolic substrates inside lysosomes, which indicated there might be no fusion barrier between lysosomes and autophagosomes in Atp6v1b2^Arg506*/Arg506*^ mice. The degradation of lysosomes depends on the presence of multiple hydrolases, and the activity of these hydrolases requires an acidic environment (De Duve, 1957; Saftig and Haas, 2016). We found abnormal degradation of metabolic substrates in lysosomes of the SGNs in Atp6v1b2^Arg506*/Arg506*^ mice as a result of the abnormal acidic environment and the affected activity of the hydrolases in the lysosomes. In addition, redundant autophagosomes were observed outside the lysosomes in the SGNs of *Atp6v1b2*^Arg50*/Arg506*^ mice, indicating that high levels of undegraded substrates in the lysosomes affected the normal process of autophagic flow (Colacurcio and Nixon, 2016).

We observed an increase in caspase-3, the ultimate initiator of apoptosis, in the SGNs and Schwann cells of Atp6v1b2^Arg506*/Arg506*^ mice, which suggested that lysosomal dysfunction induced apoptosis. Mitochondrial dysfunction and premature termination of cell cycle occur when a series of lysosomal functional abnormalities present, such as increased intraluminal pH value, abnormal degradation, and excessive accumulation of exogenous substrate for lysosomes (Hughes and Gottschling, 2012; Molin and Demir, 2014; Ruckenstuhl et al., 2014). Cell damage triggers degradation of the Bcl-2 family of proteins, which in turn activates Bax. Activated Bax/Bak complexes bind to and reduce the permeability of the mitochondrial membrane, resulting in cytochrome C release to the cytosol. Cytochrome C induces apoptosome formation, which can directly activate caspase-3 (Kroemer et al., 2007; Galluzzi et al., 2012; Ilmarinen et al., 2014).

Next, we evaluated whether treatment with the Bax inhibitor BIP-V5 could ameliorate the hearing loss in the mutant mice. After 36-week systemic administration of BIP-V5, auditory function and pathological changes improved in two out of four Atp6v1b2^Arg506*/Arg506*^-BIP-V5 mice, which further verifies that apoptosis induces the degeneration of SGNs and further impaired the hearing. As the reason for mortality was unknown in 50% (four out of eight) of the treated mice, the safety of BIP-V5 treatment needs further exploration. Bax plays an important role in programmed cell death or apoptosis and contributes to maintenance of normal psychological functions of various organs. *Atp6v1b2* gene is highly expressed in the inner ear and central nervous system of mice (Yue et al., 2014), and we presented the Arg506^∗^ mutation might induce Bax-mediated apoptosis. Therefore, administration of BIP-V5 to inhibit Bax is a reasonable way to maintain normal apoptosis in the inner ear and central nervous system. However, *Atp6v1b2* gene is rarely expressed in some organs such as the adrenal gland, intestine, stomach, liver, etc. and the mutation has minimal effect on Bax. In this case, intervention of Bax is likely to disrupt the normal physiological functions of these organs and lead to abnormalities (Kanauchi et al., 2002; Anagnostopoulos et al., 2005; Zhou et al., 2017; Al Humayed et al., 2020). Therefore, although intraperitoneal administration can control the apoptosis of spiral ganglion cells to a certain extent, it also has serious side effects on other important organs, and even leads to death. Topical administration of the inner ear might avoid the high mortality caused by intraperitoneal administration in mice, but this also brings an additional problem: the mice require long-term drug intervention, and routine operation of multiple cochlear administrations in mice can damage the structure of the inner ear, thus affecting hearing. Therefore, in order to achieve the purpose of hearing recovery by targeted drug intervention in the inner ear of mice, it is crucial to explore a new surgical method in the future.

Patients with *ATP6V1B2-*related syndromes showed severe congenital sensorineural hearing loss, which was not consistent with the milder hearing phenotype of Atp6v1b2^Arg506*/Arg506*^mice despite the presence of the same type of mutation (Menendez et al., 2017). Based on the finding that hair cells were not affected in Atp6v1b2^Arg506*/Arg506*^ mice, we hypothesized the presence of genetic compensation. Genetic compensation was already shown between isoforms of V-ATPases. For example, compensation exists between *Atp6v1g1* and *Atp6v1g2*, both of which encode the V1G subunit. The V1G subunit plays an important role in the nervous system. Defects in the V1G subunit can lead to cognitive disorders. However, *Atp6v1g2*^–/–^ mice did not show any developmental defects or obvious behavioral abnormalities until adulthood, which is considered to be associated with the upregulation of *Atp6v1g1* in the mouse brain (Kawamura et al., 2015).

The V1B subunits of the V-ATPase are encoded by *Atp6v1b2* and *Atp6v1b1*. *Atp6v1b2* mRNA is ubiquitously expressed in mouse tissues, including the inner ears. *Atp6v1b1* is mainly expressed in the kidney, epididymis, eyes, and inner ears (Tian et al., 2017). The B2 subunit of V-ATPase was shown to compensate for the function of the B1 subunit of V-ATPase in the renal medullary intercalated cells of B1-deficient mice (Paunescu et al., 2007). In this study, we observed increased transcription of *Atp6v1b1* in hair cells of Atp6v1b2^Arg506*/Arg506*^ mice and inferred that *Atp6v1b1* compensated for the *Atp6v1b2* dysfunction by increasing its own transcription level. Genetic compensation in hair cells explains the milder hearing impairment in Atp6v1b2^Arg506*/Arg506*^ mice. The mechanism of elevated transcription may be due to an enhancement in the promoter region of the gene by trimethylation (El-Brolosy et al., 2019; Ma et al., 2019). However, if a mutation occurs only at the transcriptome level and not at the DNA level, this compensatory mechanism will not be activated (Rossi et al., 2015; Ma et al., 2019). This finding was verified by our previous work: when we injected morpholinos designed to knock down *Atp6v1b2* mRNA in the cochlea of mice, the hair cells were damaged, and the mice showed severe hearing loss (Yuan et al., 2014). In addition, the cognitive and memory impairment in Atp6v1b2^Arg506*/Arg506*^mice was verified to be caused by apoptosis in the hippocampus (Supplementary Figure 8). We speculated that the function of *Atp6v1b2* gene is more important or more specific both in the SGNs and the central nervous system than in the hair cells, and thus it might not be fully compensated by other genes, or that defect of *Atp6v1b2* does not induce functional compensation in the regions of SGNs and hippocampus.

## Conclusion

**FIGURE 6:**
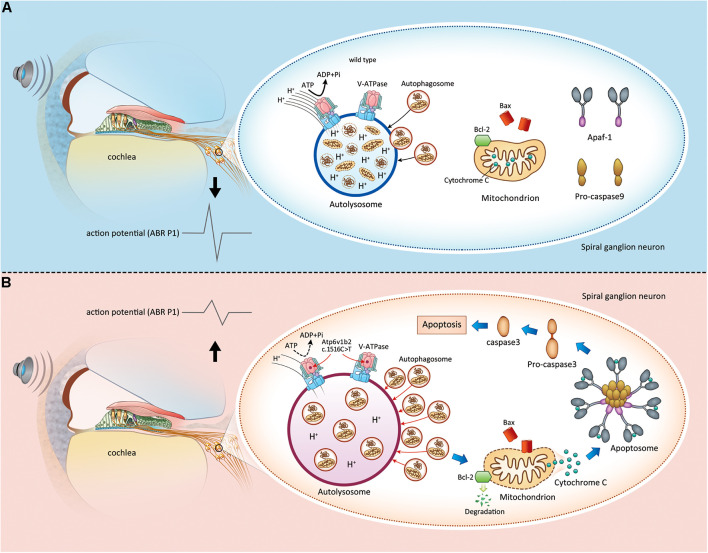
Apoptosis induced by lysosome dysfunction and autophagic flux block in spiral ganglion neurons (SGNs) causes hearing impairment in *Atp6v1b2*^Arg506*/Arg506*^ mice. **(A)** In WT mice, V-ATPases in the SGNs maintain the acidic environment by pumping protons into the lumen of lysosomes. It is a process that requires ATP hydrolysis. When autophagosomes enter the lysosomes, the intracellular components carried by autophagosomes are degraded. The magnitude of peak I (P1) amplitudes of ABR correlates with the number and synchronous firing rate of the SGN fibers. **(B)** *Atp6v1b2* c.1516 C > T affects the assembly of V-ATPases and their role in pumping protons into the lumen of lysosomes. Thus, the pH in the lysosomes increases, and the activity of acid hydrolases decreases. Under this circumstance, the autophagosomes are not effectively degraded and they accumulate in the lysosomes, which further affects the entrance of other autophagosomes. When the anti-apoptotic member of Bcl-2 family, Bcl-2, detects the acid environment disequilibrium in the lysosomes and autophagosome accumulation within the cytoplasm, it itself degrades, which in turn activates Bax/Bak complexes. Bax binds to and decreases the permeability of the mitochondrial membrane, leading to cytochrome C release. Cytochrome C binds to its partner Apaf-1 to induce the formation of a caspase-9-activating protein complex known as an apoptosome, which directly activates caspase-3, the ultimate initiator of the apoptosis process. Once apoptosis occurs in the SGNs, the hearing is impaired.

Based on the findings of this study and our previous studies, we elucidated the pathogenesis of *Atp6v1b2* defects as follows: (1) the mutation causes dysfunctional assembly between V-ATPase V1 subunits, and H^+^ transfer to lysosomes is decreased, which leads to an increased pH value; (2) disequilibrium in the acidic environment of the lysosome further affects the function of acid-dependent hydrolytic enzymes, resulting in the accumulation of degradation substrates; (3) the Bcl-2 family detects abnormal lysosome autophagy and then induces the release of cytochrome C from the mitochondria; and (4) apoptosis occurs in SGNs, affecting hearing function (Figure 6).

## Data Availability Statement

The original contributions presented in the study are included in the article/Supplementary Material, further inquiries can be directed to the corresponding author/s.

## Ethics Statement

The animal study was reviewed and approved by the institutional animal care and use committee of the Chinese PLA General Hospital.

## Author Contributions

SQ, WZ, and YY drafted the manuscript. SQ and WZ participated in the construction and analysis of *Atp6v1b2* c.1516C > T knockin mice and performed western blotting, electron microscopy, RNAscope, and BIP-V5 treatment. DL and BG carried out the experiments of immunohistochemistry. XG and WW performed the auditory evaluation and statistical analysis. YY and PD conceived the study. PC, SY, and WH participated in its design and coordination. All authors read and approved the final manuscript.

## Conflict of Interest

The authors declare that the research was conducted in the absence of any commercial or financial relationships that could be construed as a potential conflict of interest.

## Publisher’s Note

All claims expressed in this article are solely those of the authors and do not necessarily represent those of their affiliated organizations, or those of the publisher, the editors and the reviewers. Any product that may be evaluated in this article, or claim that may be made by its manufacturer, is not guaranteed or endorsed by the publisher.

## Supplementary Material

Supplementary Figure 1 *Atp6v1b2^Arg506*/Arg506*^* mice show HHL at 12 weeks and LOHL at 28 weeks. **(A)** ABR thresholds in mice aged 12 and 28 weeks. At 12 weeks, ABR thresholds showed no significant difference between WT and *Atp6v1b2^Arg506*/Arg506*^* mice. At 28 weeks, ABR thresholds of 4 and 8 kHz stimulations were significantly lower in WT than in *Atp6v1b2^Arg506*/Arg506*^* mice. **(B)** ABR P1 amplitudes in mice aged 12 and 28 weeks. *Atp6v1b2^Arg506*/Arg506*^* mice at 12 and 28 weeks had lower peaks to click as well as 4, 8, and 16 kHz stimulations than WT mice. **(C)** ABR P1 latencies in mice aged 12 and 28 weeks. At 12 weeks, *Atp6v1b2^Arg506*/Arg506*^* mice showed longer latencies to 16 and 24 kHz stimulations than WT mice. At 28 weeks, *Atp6v1b2^Arg506*/Arg506*^* mice showed significantly longer latencies to click as well as 4, 8, and 16 kHz stimulations than WT mice. The *t*-test was used to evaluate statistical significance; n represents the number of test ears, *n* = 6 for each group; ^∗^ denotes *P* < 0.05, ^∗∗^ denotes *P* < 0.01, ^∗∗∗^ denotes *P* < 0.001. Data were described as mean ± SEM (standard error of mean). **(D)** DPOAE thresholds of *Atp6v1b2^Arg506*/Arg506*^* and WT mice at 6 months after birth. The acoustic frequency was set to 4∼32kHz, and the acoustic intensity was set to gradually decrease from 80 dB SPL until reliable DPOAE signal could not be extracted. The lowest intensity of extracted DPOAE signal was taken as the threshold value of DPOAE at this frequency. The *t*-test was used to evaluate statistical significance; n represents the number of test ears, *n* = 3 for each group; *P* > 0.05. Data were described as mean ± SEM (standard error of mean). **(E)** Representative images of type II auditory nerve fibers in WT and *Atp6v1b2^Arg506*/Arg506*^* mice at high magnification. Green fluorescence represents type II auditory nerve fibers labeled by NFH (neurofilament heavy polypeptide) antibody; these nerve fibers are connected to outer hair cells. The experiments were repeated three times. **(F)** Statistical results of type II auditory nerve fibers in panel **(E)**. The *t*-test was used to evaluate statistical significance; *n* = 3 for each group. ^∗^ denotes *P* < 0.05. Data are described as mean ± SEM (standard error of mean).

Supplementary Figure 2 Ribbon synapse density is not affected in the cochlea of *Atp6v1b2^Arg506*/Arg506*^* mice. **(A–C)** Representative images of ribbon synapses immunostained with Ctbp2 (green) and DAPI (blue) in WT and *Atp6v1b2^Arg506*/Arg506*^* mice aged 4 **(A)**, 12 **(B)**, and 20 **(C)** weeks. The IHC nuclei were also labeled due to the nuclear expression of Ctbp2. The labeled dots represent ribbon synapses between IHCs and auditory nerve fibers. **(D–F)** Quantitative analysis of ribbon synapses per IHC field in the cochlea regions of 8, 16, and 32 kHz; *n* = 5 for each group. Data are described as mean ± SEM (standard error of mean). IHC: inner hair cell.

Supplementary Figure 3 *Atp6v1b2^Arg506*/Arg506*^* mice show demyelination in the osseous spiral lamina. **(A–F)** Basilar membrane of the cochlea immunostained for NFH (neurofilament heavy polypeptide, green), which preferentially stains unmyelinated axons. In the cochlea of *Atp6v1b2^Arg506*/Arg506*^* mice, ANFs (auditory nerve fibers) in the OSL (osseous spiral lamina) were labeled with NFH [**(F)**, red arrows] at 20 weeks, indicating the presence of ANF demyelination. This staining was absent in WT mice **(C)**. **(G–L)** Transmission electron microscopy of mouse cochlea. Representative images showed a few vacuoles in the myelin of *Atp6v1b2^Arg506*/Arg506*^* mice at 4 weeks [**(J)**, white arrows]. Demyelination occurred occasionally in ANFs of *Atp6v1b2^Arg506*/Arg506*^* mice at 12 weeks [**(K)**, yellow arrow]. Demyelinated ANFs were more common in *Atp6v1b2^Arg506*/Arg506*^* mice at 20 weeks [**(L)**, blue arrows]. These features were not observed in WT mice **(G–I)**. The experiments were repeated three times.

Supplementary Figure 4 No autophagy and apoptosis in the organ of Corti activated by cytochrome C were observed in *Atp6v1b2^Arg506*/Arg506*^* mice. **(A)** Representative images of immunostaining showed no increase in the number of autophagosomes labeled with LC3 in the organ of Corti of *Atp6v1b2^Arg506*/Arg506*^* mice compared with that of WT mice. **(B)** Compared with WT mice, no cytochrome C was released from the mitochondria into the cytosol in the organ of Corti of *Atp6v1b2^Arg506*/Arg506*^* mice. **(C)** No obvious cleaved caspase-3 in the organ of Corti of *Atp6v1b2^Arg506*/Arg506*^* mice was identified compared with that of WT mice.

Supplementary Figure 5 BIP-V5 was effective in improving the auditory function of *Atp6v1b2^Arg506*/Arg506*^* mice. ABR waveforms of click, 4, 8, and 16 kHz in one *Atp6v1b2^Arg506*/Arg506*^* mouse are shown. The sound intensity (dB SPL), which was used to stimulate the mice, successively decreased from high to low. The minimum sound intensity that can stimulate the mice to produce ABR waveforms was considered the hearing threshold of the mice.

Supplementary Figure 6 Western blotting analysis of the proteins of the cytochrome C-caspase-3 apoptosis pathway in the cochlea of *Atp6v1b2^Arg506*/Arg506*^* mice after BIP-V5 administration. Western blotting showed that after BIP-V5 administration, Bax was effectively inhibited and Bcl-2 level was increased, which could have prevented the permeabilization of the mitochondrial membrane and the release of cytochrome C. As a result, cleaved caspase-3 levels reduced.

Supplementary Figure 7 BIP-V5 administration increased the number of SGNs in the cochlea of *Atp6v1b2^Arg506*/Arg506*^* mice. **(A)** The number of SGNs in the cochlea of *Atp6v1b2^Arg506*/Arg506*^* mice increased after BIP-V5 administration. **(B)** The number of LC3-labeled autophagosomes in the SGNs of *Atp6v1b2^Arg506*/Arg506*^* mice was significantly increased (white arrow) compared with that of WT mice, and no obvious decrease was observed after BIP-V5 administration. **(C)** BIP-V5 reduced the release of cytochrome C from the mitochondria of SGNs in the cochlea of *Atp6v1b2^Arg506*/Arg506*^* mice. **(D)** BIP-V5 reduced the activation of caspase-3 in SGNs of *Atp6v1b2^Arg506*/Arg506*^* mice.

Supplementary Figure 8 More pronounced positive signal for cleaved caspase-3 in the region of hippocampus of *Atp6v1b2^Arg506*/Arg506*^* mice was identified.
