# Supplementary material for: Syndromic Deafness Gene ATP6V1B2 Controls Degeneration of Spiral Ganglion Neurons Through Modulating Proton Flux
Source: Front Cell Dev Biol. 2021 Oct 21;9:742714. doi: 10.3389/fcell.2021.742714 (PMC8568048; doi:10.3389/fcell.2021.742714)
Supplement: Supplementary Figure 1 — Atp6v1b2Arg506*/Arg506* mice show HHL at 12 weeks and LOHL at 28 weeks. (A) ABR thresholds in mice aged 12 and 28 weeks. At 12 weeks, ABR thresholds showed no significant difference between WT and Atp6v1b2Arg506*/Arg506* mice. At 28 weeks, ABR thresholds of 4 and 8 kHz stimulations were significantly lower in WT than in Atp6v1b2Arg506*/Arg506* mice. (B) ABR P1 amplitudes in mice aged 12 and 28 weeks. Atp6v1b2Arg506*/Arg506* mice at 12 and 28 weeks had lower peaks to click as well as 4, 8, and 16 kHz stimulations than WT mice. (C) ABR P1 latencies in mice aged 12 and 28 weeks. At 12 weeks, Atp6v1b2Arg506*/Arg506* mice showed longer latencies to 16 and 24 kHz stimulations than WT mice. At 28 weeks, Atp6v1b2Arg506*/Arg506* mice showed significantly longer latencies to click as well as 4, 8, and 16 kHz stimulations than WT mice. The t-test was used to evaluate statistical significance; n represents the number of test ears, n = 6 for each group; ∗ denotes P < 0.05, ∗∗ denotes P < 0.01, ∗∗∗ denotes P < 0.001. Data were described as mean ± SEM (standard error of mean). (D) DPOAE thresholds of Atp6v1b2Arg506*/Arg506* and WT mice at 6 months after birth. The acoustic frequency was set to 4∼32kHz, and the acoustic intensity was set to gradually decrease from 80 dB SPL until reliable DPOAE signal could not be extracted. The lowest intensity of extracted DPOAE signal was taken as the threshold value of DPOAE at this frequency. The t-test was used to evaluate statistical significance; n represents the number of test ears, n = 3 for each group; P > 0.05. Data were described as mean ± SEM (standard error of mean). (E) Representative images of type II auditory nerve fibers in WT and Atp6v1b2Arg506*/Arg506* mice at high magnification. Green fluorescence represents type II auditory nerve fibers labeled by NFH (neurofilament heavy polypeptide) antibody; these nerve fibers are connected to outer hair cells. The experiments were repeated three times. (F) Statistical results of type II a [file Data_Sheet_1.PDF]

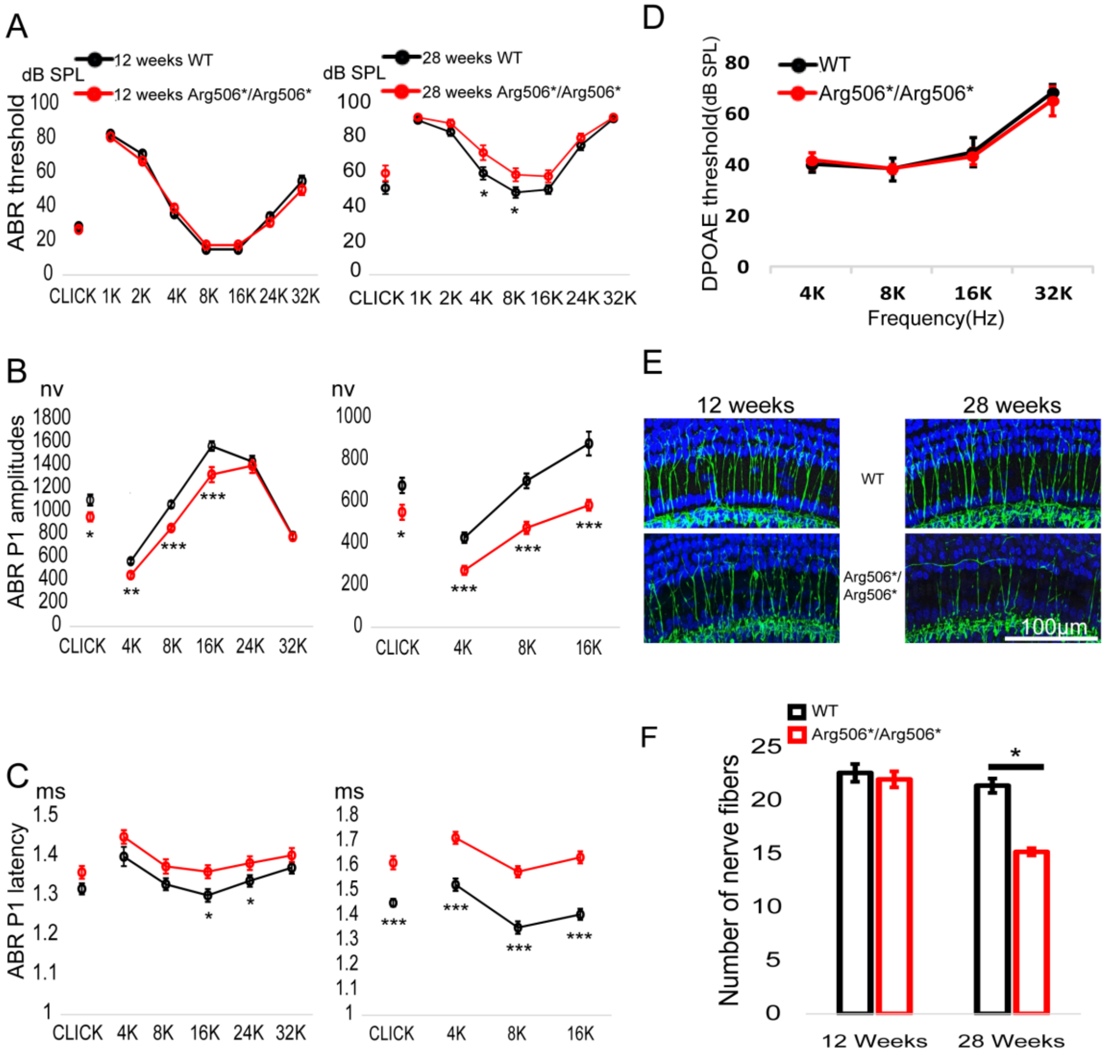

Figure S1. *Atp6v1b2*$^{Arg506*/Arg506*}$ mice show HHL at 12 weeks and LOHL at 28 weeks.

(A) ABR thresholds in mice aged 12 and 28 weeks. At 12 weeks, ABR thresholds showed no significant difference between WT and *Atp6v1b2*$^{Arg506*/Arg506*}$ mice. At 28 weeks, ABR thresholds of 4 and 8 kHz stimulations were significantly lower in WT than in *Atp6v1b2*$^{Arg506*/Arg506*}$ mice. **(B)** ABR P1 amplitudes in mice aged 12 and 28 weeks. *Atp6v1b2*$^{Arg506*/Arg506*}$ mice at 12 and 28 weeks had lower peaks to click as well as 4, 8, and 16 kHz stimulations than WT mice. **(C)** ABR P1 latencies in mice aged 12 and 28 weeks. At 12 weeks, *Atp6v1b2*$^{Arg506*/Arg506*}$ mice showed longer latencies to 16 and 24 kHz stimulations than WT mice. At 28 weeks, *Atp6v1b2*$^{Arg506*/Arg506*}$ mice showed significantly longer latencies to click as well as 4, 8, and 16 kHz stimulations than WT mice. The t-test was used to evaluate statistical significance; n represents the number of test ears, n=6 for each group; * denotes P<0.05, ** denotes P<0.01, *** denotes P<0.001. Data were described as mean ± SEM (standard error of mean). **(D)** DPOAE thresholds of *Atp6v1b2*$^{Arg506*/Arg506*}$ and WT mice at 6 months after birth. The acoustic frequency was set to 4 kHz~32kHz, and the acoustic intensity was set to gradually decrease from 80 dB SPL until reliable

DPOAE signal could not be extracted. The lowest intensity of extracted DPOAE signal was taken as the threshold value of DPOAE at this frequency. The t-test was used to evaluate statistical significance; n represents the number of test ears, n=3 for each group; P>0.05. Data were described as mean ± SEM (standard error of mean). **(E)** Representative images of type II auditory nerve fibers in WT and *Atp6v1b2*$^{Arg506*/Arg506*}$ mice at high magnification. Green fluorescence represents type II auditory nerve fibers labeled by NFH (neurofilament heavy polypeptide) antibody; these nerve fibers are connected to outer hair cells. The experiments were repeated three times. **(F)** Statistical results of type II auditory nerve fibers in Figure S1E. The t-test was used to evaluate statistical significance; n=3 for each group. * denotes P<0.05. Data are described as mean ± SEM (standard error of mean).

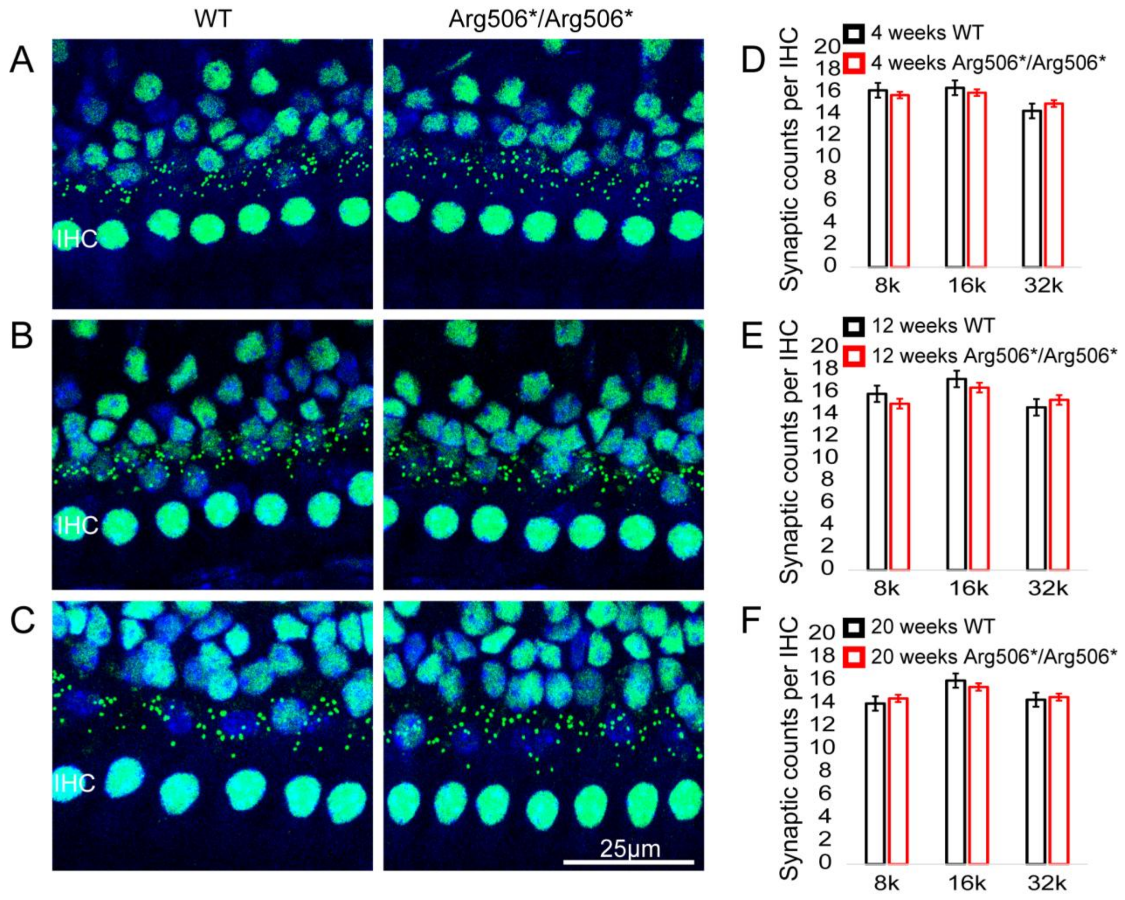

Figure S2. Ribbon synapse density is not affected in the cochlea of *Atp6v1b2*$^{Arg506*/Arg506*}$ mice.

(A, B, C) Representative images of ribbon synapses immunostained with Ctbp2 (green) and DAPI (blue) in WT and *Atp6v1b2*$^{Arg506*/Arg506*}$ mice aged 4 **(A)**, 12 **(B)**, and 20 **(C)** weeks. The IHC nuclei were also labeled due to the nuclear expression of Ctbp2.The labelled dots represent ribbon synapses between IHCs and auditory nerve fibers. **(D, E, F)** Quantitative analysis of ribbon synapses per IHC field in the cochlea regions of 8, 16, and 32 kHz; n=5 for each group. Data are described as mean ± SEM (standard error of mean). IHC: inner hair cell.

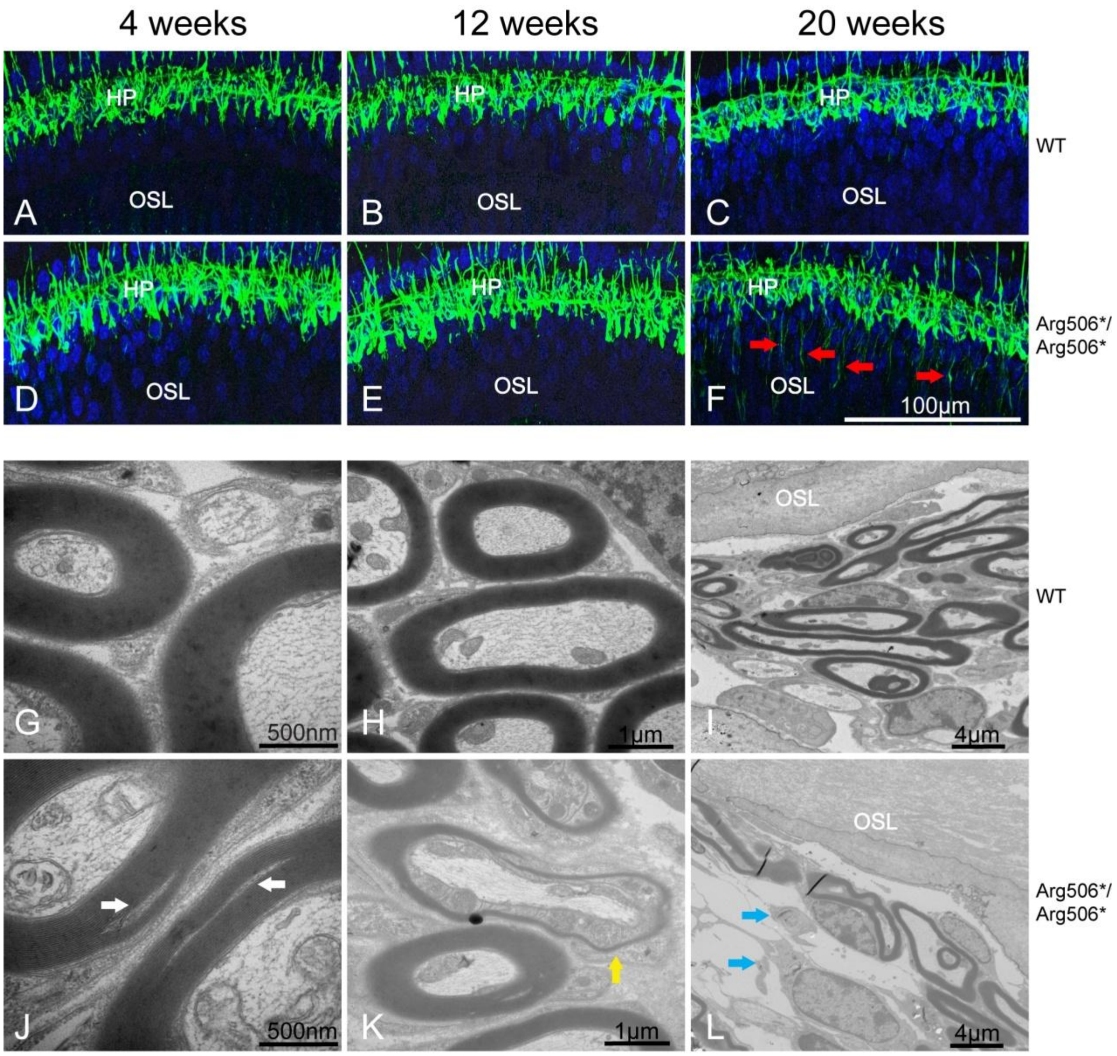

Figure S3. ***Atp6v1b2*** $^{\mathbf{Arg506*/Arg506*}}$ **mice show demyelination in the osseous spiral lamina.**

(A-F) Basilar membrane of the cochlea immunostained for NFH (neurofilament heavy polypeptide, green), whichpreferentially stainsunmyelinated axons.In the cochlea of *Atp6v1b2*$^{Arg506*/Arg506*}$ mice, ANFs (auditory nerve fibers) in the OSL (osseous spiral lamina) were labeled with NFH (F, red arrows) at 20 weeks, indicating the presence of ANF demyelination. This staining was absent in WT mice (C). **(G-L)** Transmission electron microscopy of mouse cochlea. Representative images showed a few vacuoles in the myelin of *Atp6v1b2*$^{Arg506*/Arg506*}$ mice at 4 weeks (J, white arrows). Demyelination occurred occasionally in ANFs of *Atp6v1b2*$^{Arg506*/Arg506*}$ mice at 12 weeks (K, yellow arrow). Demyelinated ANFs were more common in *Atp6v1b2*$^{Arg506*/Arg506*}$ mice at 20 weeks (L, blue arrows). These features were not observed in WT mice (G, H, I). The experiments were repeated three times.

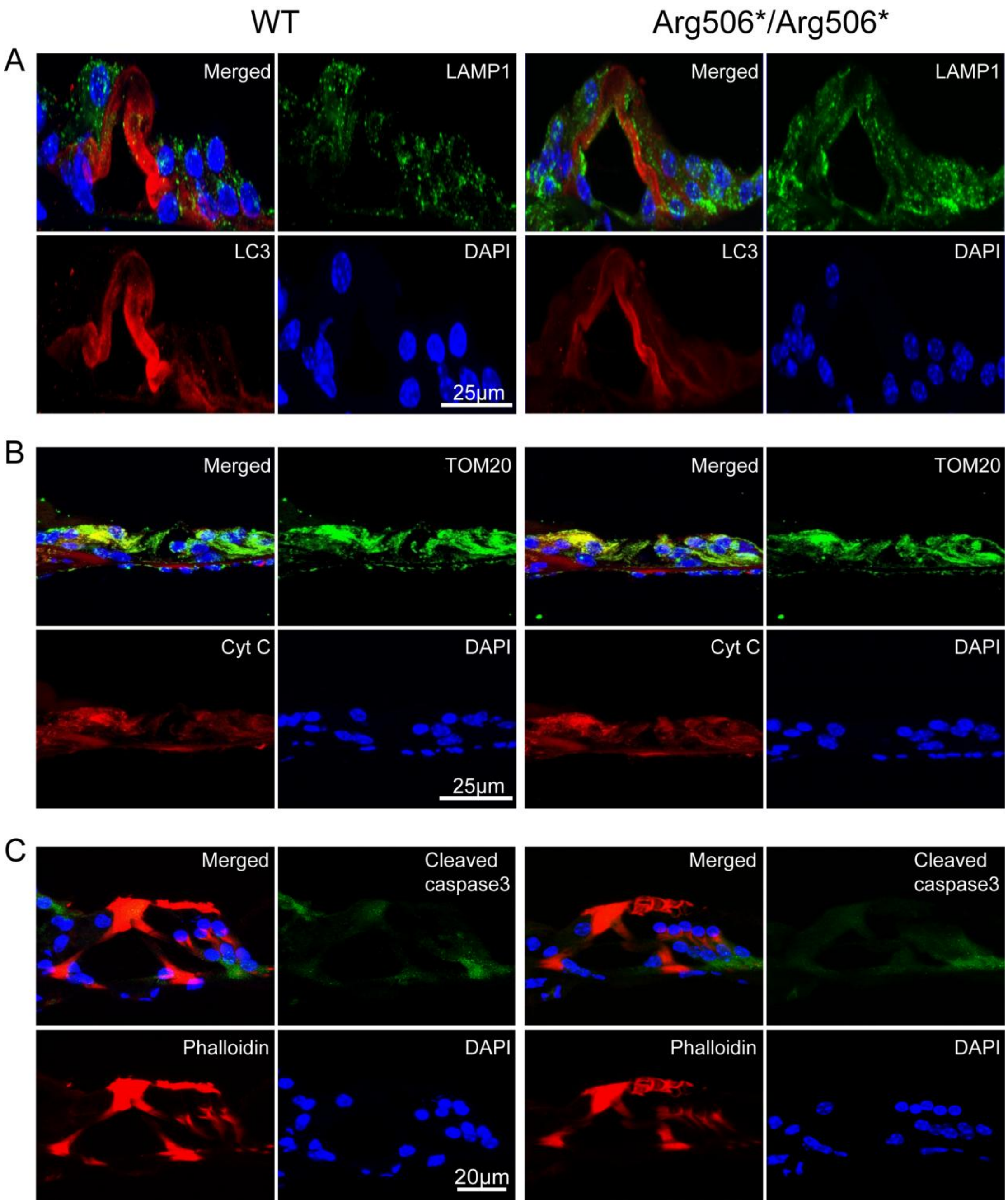

Figure S4. No autophagy and apoptosis in the organ of Corti activated by cytochrome C were observed in *Atp6v1b2*$^{\mathbf{Arg506*/Arg506*}}$ mice.

(A) Representative images of immunostaining showed no increase in the number of autophagosomes labeled with LC3 in the organ of Corti of *Atp6v1b2*$^{Arg506*/Arg506*}$ micecompared with that of WT mice. **(B)** Compared with WT mice, no cytochrome C wasreleased from the mitochondria into the cytosol in the organ of Corti of *Atp6v1b2*$^{Arg506*/Arg506*}$ mice. **(C)** No obvious cleaved caspase-3 in the organ of Cortiof *Atp6v1b2*$^{Arg506*/Arg506*}$ mice was identified compared with that of WT mice.

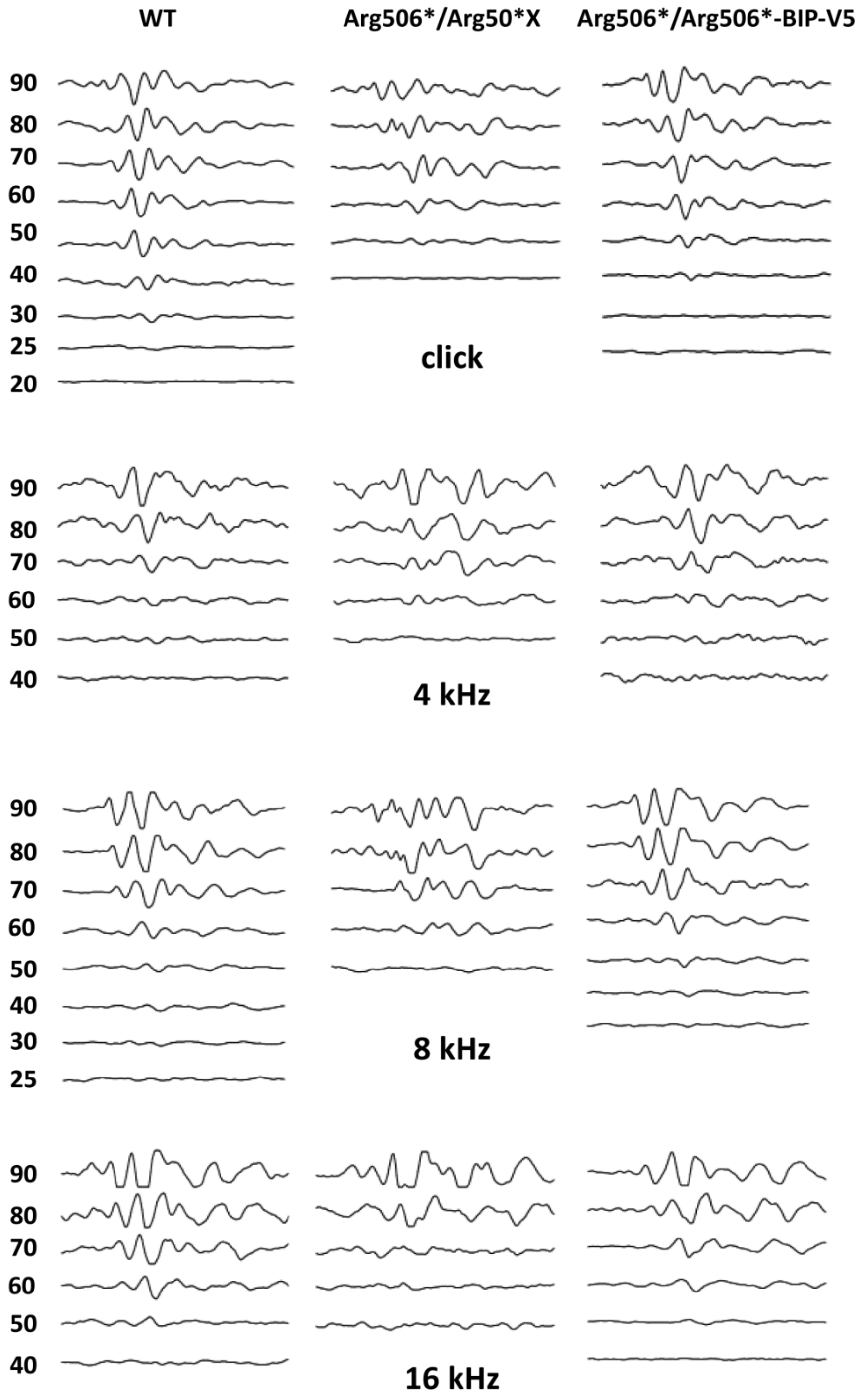

Figure S5. BIP-V5 was effective in improving the auditory function of *Atp6v1b2*$^{Arg506*/Arg506*}$ mice.

ABR waveforms of click, 4 kHz, 8 kHz, and 16 kHzin one *Atp6v1b2*$^{Arg506*/Arg506*}$ mouse are shown. The sound intensity (dB SPL), which was used to stimulate the mice, successively decreased from high to low. The minimum sound intensity that can stimulate the mice to produce ABR waveforms was considered the hearing threshold of the mice.

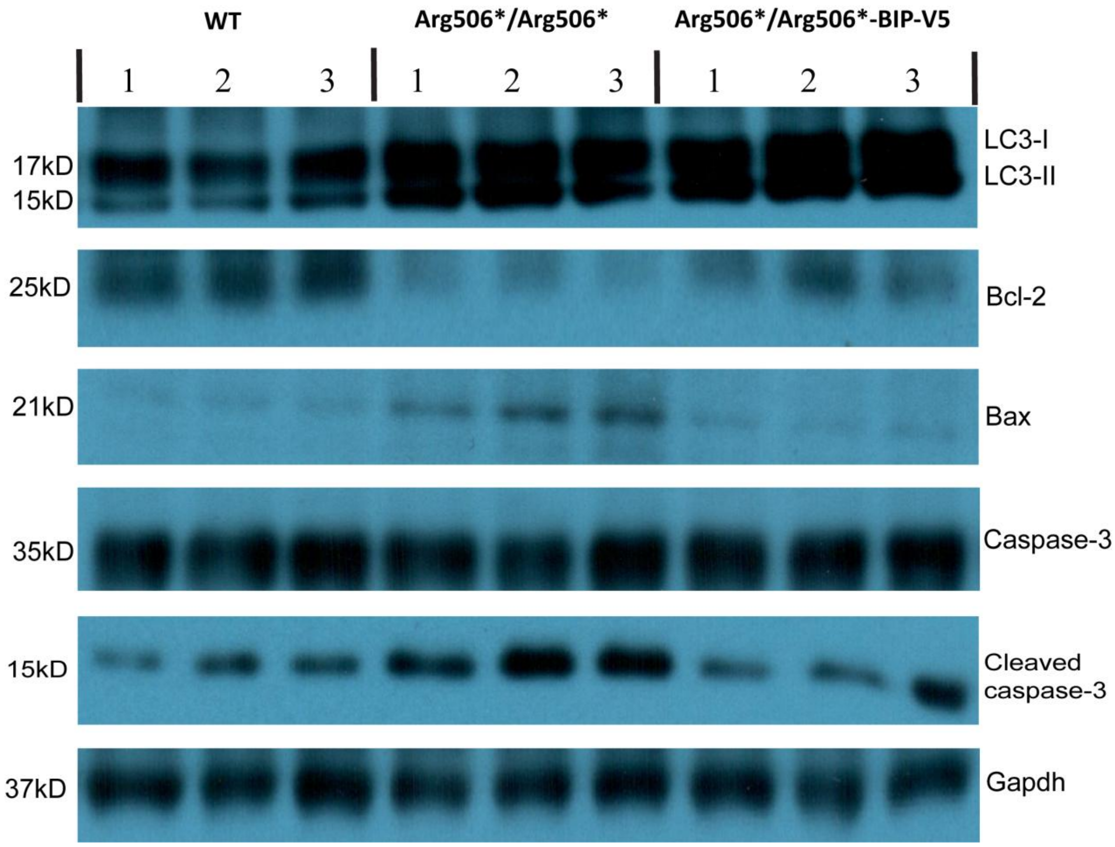

Figure S6. Western blotting analysis of the proteins of the cytochrome C-caspase-3 apoptosis pathway in the cochlea of *Atp6v1b2*$^{\mathbf{Arg506*/Arg506*}}$ mice after BIP-V5 administration.

Western blotting showed that after BIP-V5 administration, Bax was effectively inhibited and Bcl-2 level was increased, which could have prevented the permeabilization of the mitochondrial membrane and the release of cytochrome C. As a result, cleaved caspase-3 levels reduced.

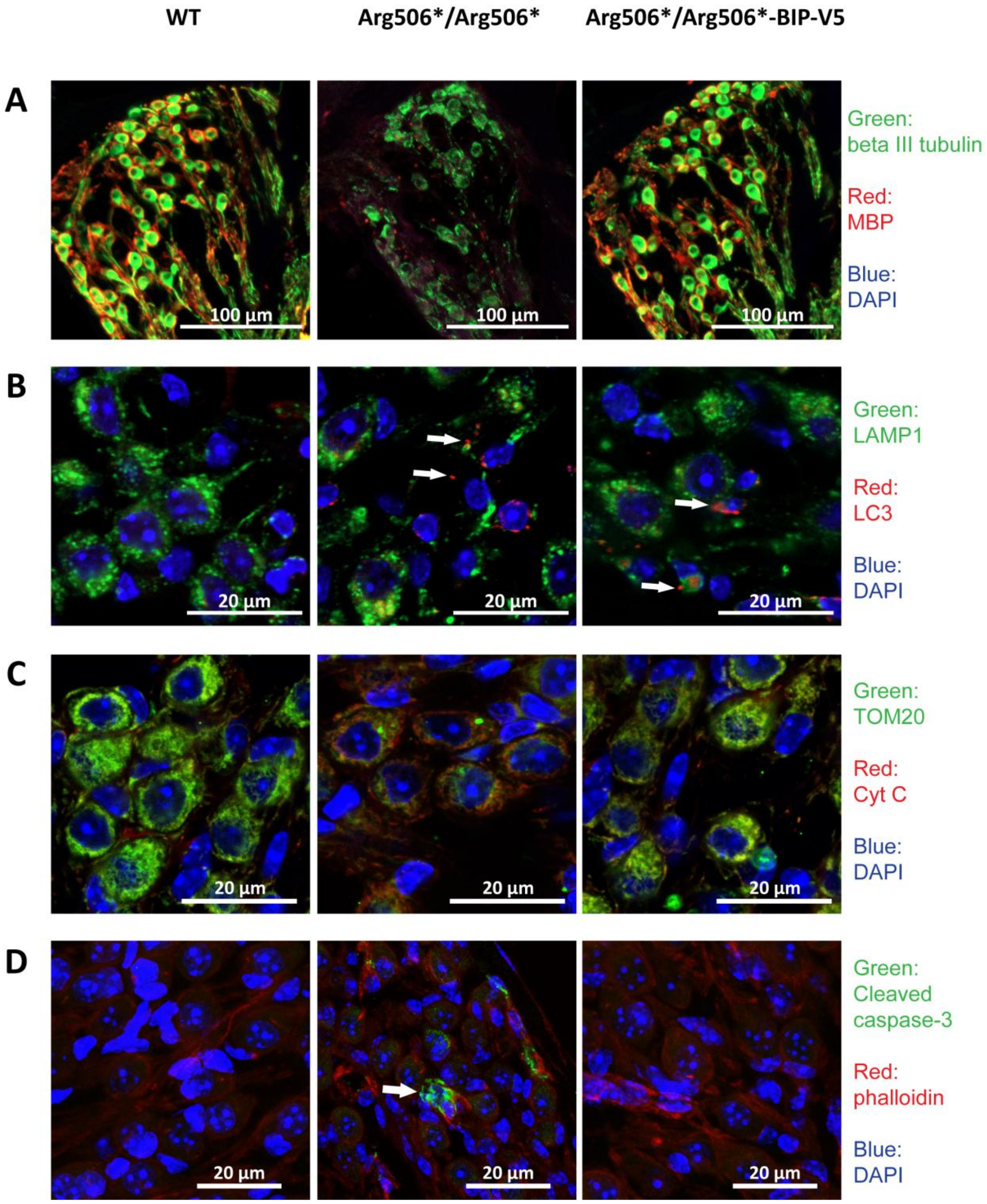

Figure S7. BIP-V5 administration increased the number of SGNs in the cochlea of *Atp6v1b2*$^{\mathrm{Arg506*/Arg506*}}$ mice.

(A) The number of SGNs in the cochlea of *Atp6v1b2*$^{\mathrm{Arg506*/Arg506*}}$ mice increased after BIP-V5 administration. **(B)** The number of LC3-labeled autophagosomes in the SGNs of *Atp6v1b2*$^{\mathrm{Arg506*/Arg506*}}$ mice was significantly increased (white arrow) compared with that of WT mice, and no obvious decrease was observed after BIP-V5 administration. **(C)** BIP-V5 reduced the release of cytochrome C from the mitochondria of SGNs in the cochlea of *Atp6v1b2*$^{\mathrm{Arg506*/Arg506*}}$ mice. **(D)** BIP-V5 reduced the activation of caspase-3 in SGNs of *Atp6v1b2*$^{\mathrm{Arg506*/Arg506*}}$ mice.

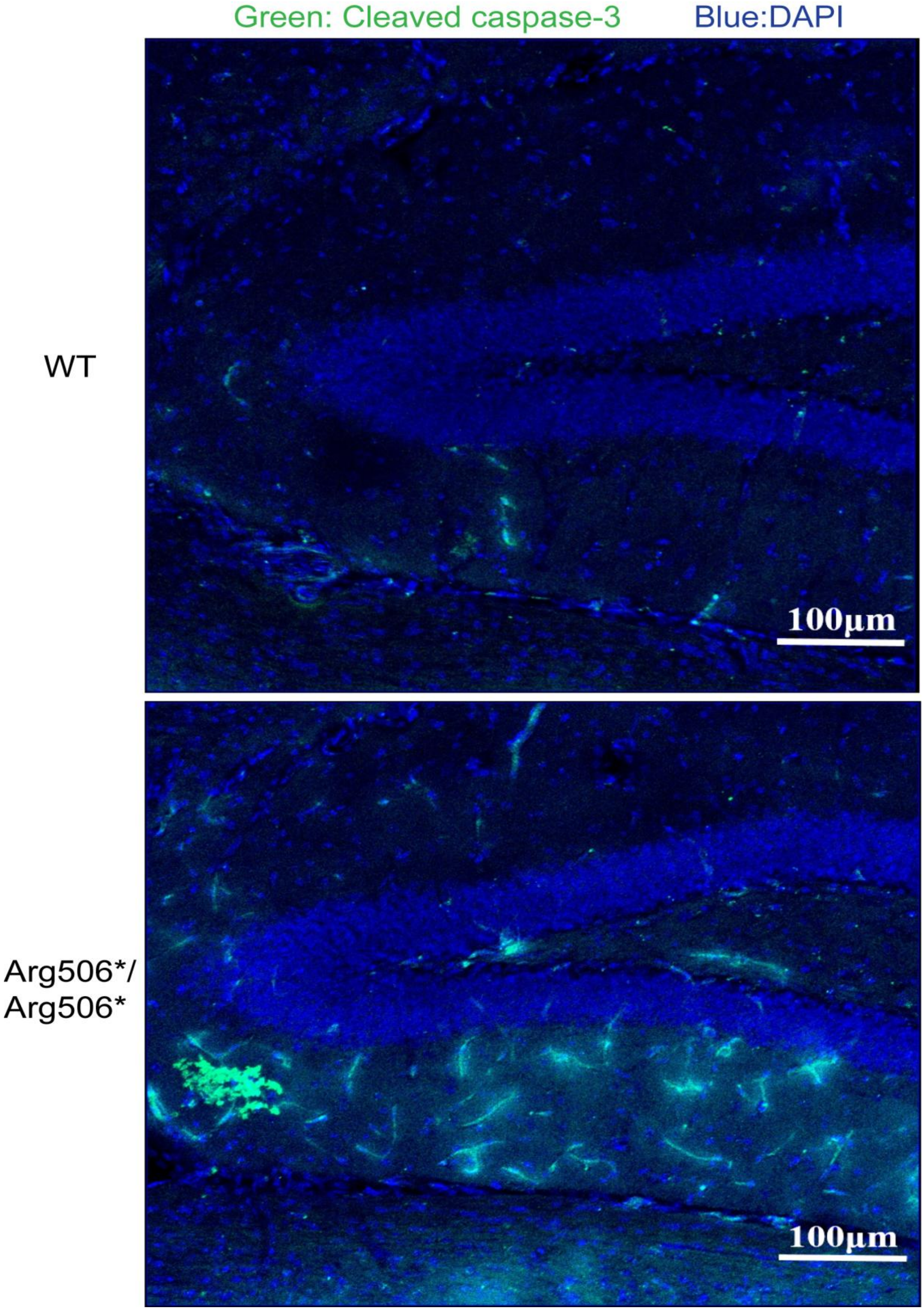

Figure S8. More pronounced positive signal for cleaved caspase-3 in the region of hippocampusof *Atp6v1b2*$^{Arg506*/Arg506*}$ mice was identified.
